# Addressing community mental health needs in the United States: a comparison of the federal Certified Community Behavioral Health Clinic and Massachusetts Community Behavioral Health Center models

**DOI:** 10.3389/frhs.2025.1681093

**Published:** 2025-11-21

**Authors:** Maxim Petrovsky, Swathi Damodaran, Christopher T. Lim

**Affiliations:** 1 Boston University Chobanian & Avedisian School of Medicine, Boston, MA, United States; 2 Office of Clinical Affairs, University of Massachusetts Chan Medical School ForHealth Consulting, Shrewsbury, MA, United States; 3 Department of Psychiatry, Boston University Chobanian & Avedisian School of Medicine, Boston, MA, United States; 4 Population Health Services, Boston Medical Center Health System, Boston, MA, United States

**Keywords:** community behavioral health, value based care, community behavioral health centers (CBHCs), certified community behavioral health clinics (CCBHCs), outpatient mental health, quality incentive program, medicaid

## Abstract

The United States has long sought to create a scalable community mental health and substance use continuum. Federal Certified Community Behavioral Health Clinics (CCBHC) have grown to be the dominant model for comprehensive community mental health services across the US since 2014. In parallel, the state of Massachusetts established its Community Behavioral Health Center (Massachusetts CBHC) model, going live in 2023. Central to both models is a foundational outpatient clinic offering multidisciplinary health and social services that utilizes a bundled payment structure—typically a day-rate or, in some cases for CCBHCs, a monthly rate—eschewing a traditional fee-for-service payment structure. These models differ in other aspects of their clinical models, federal financial support, and provider payment mechanisms.

## Introduction

An effective model of a comprehensive behavioral health continuum in the United States has been elusive for decades following deinstitutionalization in the mid-20th century. With the more recent trends of rising awareness of mental health needs across the US population, destigmatization, and the opioid crisis, a renewed federal and state focus on mental health has led to legislative actions toward a foundation of this vision. Across the US, the Certified Community Behavioral Health Clinic (CCBHC) has been the dominant model that has emerged from the federal government to meet this need, bolstered by substantial and continued federal investment (1). However, the implementation of the CCBHC model is not consistent across all parts of the country, with large variation seen state by state given the mechanisms of the federal policies and funding of CCBHCs. In Massachusetts, a similar but distinct model called the Community Behavioral Health Center (CBHC) launched in 2023. This paper describes and compares the federal CCBHC and Massachusetts CBHC models, including the policy background, clinical models, funding and payment models, and provider quality incentives for each (Table 1). We discuss the implications of the many similarities and few differences, and make suggestions for the evolving community behavioral health landscape.

**Table 1:** Overview of CCBHC and Massachusetts CBHC models.

Dimension	Federal CCBHC	Massachusetts CBHC
Clinical model	Nine core services: 24 h crisis care, screening/assessment, person-/family-centered treatment planning, outpatient mental health & substance use disorder treatment, primary care screening, targeted case management, psychiatric rehabilitation, peer/family support, and intensive services for veterans.	Rapid access (same-/next-day appointments), 24/7 crisis services including mobile crisis intervention and community crisis stabilization, evidence-based treatments (e.g., Dialectical Behavior Therapy, Cognitive Behavioral Therapy), integrated physical/behavioral health care, and social supports.
Federal funding	Eligible for an enhanced federal match (eFMAP) under the demonstration program, with 100% FMAP for tribal facilities; additional SAMHSA expansion grants available.	Receives a base federal match of 50% (Massachusetts's standard FMAP rate), relying on additional state funding or an 1,115 waiver to support program innovations.
Provider payment mechanism	Uses a cost-based Prospective Payment System (PPS) (CC PPS-1 to CC PPS-4), which can be daily or monthly. Each clinic's rate is determined by its actual costs; includes annual updates and scheduled rebasing.	Relies on encounter bundle rates for core outpatient services and fee-for-service for crisis and specialty services. Rates are uniform across all CBHCs, rather than individualized by clinic costs.
Quality incentives	May offer Quality Bonus Payments (QBPs) for meeting or exceeding performance benchmarks, depending on the PPS selected by the state. States have flexibility in defining thresholds, payment methodologies, and measures.	Includes a mandatory Clinical Quality Incentive Program (CCQI) and the Quality and Equity Incentive Program (CQEIP) focusing on access, crisis stabilization, and equity.

## Policy background on CCBHCs and Massachusetts CBHCs

The national Certified Community Behavioral Health Clinic (CCBHC) model was created with the passage of the Protecting Access to Medicare Act (PAMA) of 2014 in section 223 of the statute. This led the US Department of Health and Human Services (HHS) to conduct a two-phase implementation, with a one-year planning grant to 24 states followed by a two-year Medicaid demonstration program for eight of those 24 states (2). The ultimate goal of this demonstration program was to create a comprehensive, ambulatory behavioral health model paid through a new prospective payment system (PPS) to improve the availability, quality, and outcomes of behavioral health services focused on individuals with serious mental illnesses as well as co-occurring substance use disorders. Since that time, section 223 has been extended and expanded, first in 2020 with the addition of two states to the demonstration program through the Coronavirus Aid, Relief, and Economic Security Act (CARES Act), and again in 2022 with section 11,001 of the Bipartisan Safer Communities Act to add an additional ten states every two years beginning in 2024 (3).

In addition to the demonstration programs paid for through the PPS, the Substance Abuse and Mental Health Services Administration (SAMHSA) has provided expansion funding to several non-demonstration and demonstration behavioral health clinics around the United States (4). This funding has taken the form of expansion grants over one or two years. All-in-all, there are over 500 demonstration and expansion CCBHCs in the United States as of this writing (5).

While CCBHC demonstration programs have continued to spread across the United States, some states have sought to pursue other methods of behavioral health clinic expansion, such as through 1,115 Medicaid waivers, state plan amendments, or state legislation.

In Massachusetts, leaders across the state's Executive Office of Health and Human Services (EOHHS) also developed a comprehensive system of behavioral health services across the state. The intention was to create an easy-access “front door to treatment” inclusive of crisis supports and outpatient treatment (6–8). Massachusetts Community Behavioral Health Centers (CBHCs) were developed as part of the broader Roadmap for Behavioral Health Reform, based on listening sessions in 2019, introduced in 2021, and implemented in 2023, with the goal of expanding access to more community-based mental health and substance use treatment in the state. These listening sessions, which highlighted the challenges and gaps in the behavioral health system, combined with the state not having been chosen as one of the original CCBHC demonstration states, as well as the COVID-19 pandemic exacerbating many longstanding issues with the behavioral health system, led Massachusetts to pursue the development of CBHCs as part of this Roadmap. Twenty-five Community Behavioral Health Centers (CBHCs) launched in 2023 (9, 10).

## CCBHC and Massachusetts CBHC clinical models

CCBHCs are required to provide a comprehensive scope of services for individuals with serious mental illness or co-occurring substance use disorder. These include nine core services, four of which must be provided by the CCBHC and five others that may be provided by CCBHCs or through contracted providers known as designated collaborating organizations. The four services required to be directly provided are (1) 24 h crisis services (including mobile crisis teams), (2) screening, assessment, and diagnosis, (3) person- and family-centered treatment planning, and (4) outpatient mental health and substance use treatment. The five services that may be provided by a designated collaborating organization include (5) outpatient primary care screening and monitoring, (6) targeted case management services, (7) psychiatric rehabilitation services, (8) peer and family/caregiver support and counseling, and (9) intensive, community-based mental health care for members of the armed forces and veterans (11). All federal demonstration or expansion grant CCBHCs are required to meet a set of certification criteria across six categories put forth by SAMHSA and HHS (12).

The CCBHC model has demonstrated early favorable results, although analyses have been limited by short time scales and small study populations. A 2022 performance analysis of three original CCBHC demonstration program states—Missouri, Oklahoma, and Pennsylvania—found some promising results in regard to behavioral health crisis and acuity over the first two years, FY 2018 and FY 2019. Only in Oklahoma did the authors find a significant decrease in the probability of an inpatient hospitalization for any reason following CCBHC implementation, while in Oklahoma and Pennsylvania they found decreases in behavioral health-related emergency department (ED) visits. In Missouri, researchers found no significant change in inpatient hospitalizations nor behavioral health ED visits (13). Of note, in Oklahoma, there was a significantly higher probability of an any-cause ED visit in the CCBHC implementation period. In aggregate across all seven CCBHC demonstration states, all-cause hospital readmissions decreased from 22% to 16% between year 1 and year 2. This same study found increases in the evidence-based practices offered by providers, including a 46% increase in 24 h mobile crisis teams, 27% increase in supported employment, and an increase in supported housing of 12% (13). A later study of the same three states, Missouri, Oklahoma, and Pennsylvania, during the same time period, echoed these results, showing that CCBHC beneficiaries in Pennsylvania and Oklahoma had significant reductions in the average number of behavioral health-related ED visits, with no change found in Missouri. While not statistically significant, it was found that CCBHCs in Oklahoma were associated with a 22% decrease in all-cause hospitalizations over two years (14).

In Massachusetts, CBHCs are tasked with providing timely, accessible care for mental health and substance use disorders, emphasizing urgent access, crisis stabilization services, integrated services, and recovery-oriented care. They offer same-day or next-day appointments and crisis care, individual, family, and group therapy, medication for mental health and substance use disorders, and mobile crisis services in the community. These services are provided through three main components: (1) core outpatient services, (2) mobile crisis, and (3) community crisis stabilization. Of these three, community crisis stabilization differs the most significantly from the CCBHC model, offering a 24/7 alternative to inpatient psychiatric hospitalization in a dedicated unit. This is a service intended for both children and adults who may be in psychiatric crisis and need higher-acuity care but are not suicidal or homicidal.

In addition, CBHCs integrate behavioral and physical health through medical screenings and basic physical health care. Care must be coordinated across this spectrum of services and broad social supports including housing, social services, and employment supports through partnerships with other health care and social service agencies. Recovery is central to the CBHC model, incorporating peer and family supports, as well as extended hours, 24/7 crisis support, telehealth options, and community-based care (15).

## Federal match for CCBHC and Massachusetts CBHC programs

A key component of the CCBHC demonstration program being a Medicaid benefit is the federal match. The federal match for Medicaid, known as the Federal Medical Assistance Percentage (FMAP), is the rate at which the federal government supports states for Medicaid expenditures, varying by state based on per capita income, ranging from 50% to 83%. States with lower incomes receive a higher federal match. In addition, there are Enhanced Federal Medical Assistance Percentages (eFMAP) that support the Children's Health Insurance Program (CHIP) under Title XXI of the Social Security Act (16).

CCBHCs under the federal demonstration receive an enhanced federal match rate equivalent to the CHIP eFMAP match rate for the array of CCBHC medical assistance services for 16–24 quarters (4–6 years), depending on when the enrollee state was added to the demonstration program. This enhanced match is provided to incentivize states to implement the CCBHC model and support the more comprehensive and integrated services entailed. Under the authority of PAMA, states may claim the eFMAP for clinics participating in the demonstration program, without the need for a state plan amendment.

In addition, for services provided by CCBHCs that qualify as Indian Health Service or tribal facilities, the federal match rate is 100%. Expenditures for qualifying community-based mobile crisis services under the American Rescue Plan (ARP), provided by both CCBHCs and CBHCs, can receive an increased FMAP rate of 85% for the first 12 fiscal quarters within the five-year period starting April 1, 2022, and ending March 31, 2027 (17).

In contrast to the federal demonstration CCBHCs, Massachusetts CBHCs receive the base federal match rate of 50%, consistent with the standard FMAP applicable to Massachusetts Medicaid programs for all services except for crisis care. 50% is the lowest possible match percentage as mandated by federal law due to a higher per capita income in Massachusetts relative to other states. Under a potential demonstration program, the federal share would reach the eFMAP for Massachusetts of 65% (18).

## Provider payment mechanisms

Differences in how CCBHCs and Massachusetts CBHCs are paid reflect their respective policy and operational environments. Demonstration CCBHCs operate under a cost-based prospective payment system (PPS), which is designed to provide predictable funding that aligns with the cost of delivering the comprehensive array of services. This model aims to incentivize the provision of a wide range of behavioral health services, including crisis intervention, peer support, support for social determinants of health, and care coordination services, that may not be otherwise covered under a traditional fee-for-service system.

The demonstration program offers states several PPS rate-setting methodologies for CCBHCs that participating states can choose from to reimburse clinics for the expected cost of providing services. It is important to note that each individual clinic receives a separate rate depending on the associated costs of service provision. The first option is the Certified Clinic Prospective Payment System 1 (CC PPS-1), a Federally Qualified Health Center-like PPS rate that provides reimbursement on a daily basis for all CCBHC services provided on any given day. States have the option to provide Quality Bonus Payments (QBPs) to CCBHCs that meet quality performance thresholds. The Certified Clinic Prospective Payment System 2 (CC PPS-2) provides a monthly rate and allows states to develop separate Special Population (SP) rates to cover the higher costs associated with individuals with certain clinical conditions. In addition, the CC PPS-2 requires states to incorporate QBPs and outlier payments for high-cost cases. The third methodology, the Certified Clinic Prospective Payment System 3 (CC PPS-3), provides a daily rate and includes separate Special Crisis Services (SCS) rates for crisis services. SCS rates may be set for mobile crisis intervention or on-site crisis stabilization services. The fourth and final option, the Certified Clinic Prospective Payment System 4 (CC PPS-4) is a monthly unit of payment and combines required QBPs, optional special population rates for people with certain conditions, and required separate monthly SCS rates for crisis services (17).

Each of these PPS methodologies is designed to ensure that CCBHCs are adequately reimbursed for the cost of providing comprehensive, person-centered care, with an emphasis on maintaining quality and managing high-cost cases effectively. States must update PPS rates annually using the Medicare Economic Index, to keep pace with inflation, and must rebase them every three years to align payments with actual service costs using cost report data (17).

In contrast, Massachusetts CBHCs operate under a payment model which includes a combination of encounter bundle rates and fee-for-service elements. This payment model is set forth via the regulatory authority of the MA Executive Office of Health and Human Services and approved by the Centers for Medicare and Medicaid Services (CMS) (18, 19). The Massachusetts CBHC payment methods share some similarities with the CCBHC demonstration programs but differ in key ways as well.

For core outpatient clinical services, Massachusetts CBHCs use an encounter bundle rate that involves a flat, per-diem rate per patient for a set of standard, designated services, regardless of the number of services provided to an individual on that date. This rate differs depending on whether the individual is an adult or child/adolescent, but otherwise is the same for all CBHCs across the state. As with CCBHCs, this enables reimbursement to CBHCs for services that would typically not be covered by traditional fee-for-service billing, such as care coordination and support for social determinants of health (20).

In addition, Massachusetts maintains rates and billing for crisis services separate from the outpatient bundled encounter rate; these rates may be billed on the same date as bundled encounters. These crisis services include community crisis stabilization and mobile crisis intervention. Rates for these two services vary based on if the intervention is provided to an adult or youth, the qualification of the service provider, and, to some extent, the length of the service (19).

Notably, neither the federal CCBHCs nor Massachusetts CBHCs have a requirement to contract with commercial payers, nor do these payers have a requirement to participate in the per-diem rate structure, so broader accessibility of CCBHC or Massachusetts CBHC services to local patient populations varies from state-to-state and even clinic-to-clinic. Medicare covers CCBHCs and Massachusetts CBHCs but is not required to pay the bundled-payment (21). In Massachusetts, community members who are not covered by Medicaid or whose insurance does not cover CBHC services are nonetheless entitled to CBHC mobile crisis intervention and community crisis stabilization as well as three dates of core outpatient services, paid through a state trust, the Behavioral Health Access and Crisis Intervention (BHACI) Trust Fund (22). Fully-insured commercial payers in Massachusetts are required to cover behavioral health crisis intervention services including, but not limited to, mobile crisis teams, community crisis stabilization services, and urgent outpatient behavioral health treatment. Commercial payers are also encouraged to contract with CBHCs for standard, ongoing outpatient services as part of their provider network (23).

## Quality improvement payment components

Both the federal CCBHC demonstration program and the Massachusetts CBHC program incentivize providers to support good quality care and encourage the transition to value-based care through quality incentives.

CCBHC quality measurement entails both clinic- and state-collected measures, some of which are required and others that are optional. For broader national evaluation, there is a larger set of measures that include the pay-for-performance Quality Bonus Payment (QBP) measures as well as others. Measures used in the QBP program are a smaller subset of the evaluation measures and fall into two categories: clinical outcome measures and process measures focused on screening, follow-up, and risk assessment. QBP outcome measures include comprehensive diabetes care via hemoglobin A1c control, depression remission at six months, and all-cause readmissions rate. QBP process measures include, but are not limited to, time to service access, follow-up after hospitalization, initiation and engagement of substance use disorder treatment, and suicide risk assessments. QBPs are intended to encourage high performance by rewarding CCBHCs that meet or exceed quality thresholds. States can set different performance thresholds and tier the payments based on levels of achievement (17).

As mentioned previously, QBPs are optional under the CC PPS-1 and CC PPS-3 but are required for the CC PPS-2 and CC PPS-4 methodologies. To receive QBPs, CCBHCs must achieve the state-established benchmarks and do not receive payments for reporting alone. States have substantial flexibility around setting the performance threshold that triggers payment for each measure, frequency of payment, the amount of payment, and other mechanics of the payment given to providers (24).

Similarly, Massachusetts has implemented two quality programs for CBHCs: the CBHC Clinical Quality Incentive Program (CCQI) and the CBHC Quality and Equity Incentive Program (CQEIP). These represent the state's broader efforts to enhance the quality of behavioral health services and to make health equity a core pillar of behavioral health delivery. These quality programs are a five-year performance program with a total of $8.5 million available across the entire state for each program in the first performance year (25).

Broadly, Massachusetts CBHCs aim to provide quick and appropriate access to care, timely crisis stabilization, and effective care coordination. Accordingly, the CCQI measures assess CBHCs on their timeliness of access to care, their provision of care to individuals following discharge from an acute behavioral episode of care, and the rate of readmission to the same or higher level of care within 30 days of visiting a CBHC. Similar to the federal demonstration CCBHCs, Massachusetts' CCQI program has been updated per federal CMS direct payment regulations to be a pay-for-performance program throughout all quality years (26).

CQEIP is designed to improve health equity by addressing health-related social needs and disparities through the behavioral health system and CBHCs. It incentivizes CBHCs to make improvements in three domains: (1) demographic and health-related social needs data reporting, (2) equitable quality and access to services, including accommodations for those with disabilities or limited English proficiency, and (3) capacity and collaboration related to workforce and collaboration with other health system partners. All CQEIP measures are pay-for-performance throughout the quality program (26).

Both the Massachusetts CCQI and CQEIP programs feature interim estimated payments, followed by a final reconciliation or recoupment depending on performance outcomes.

## Discussion

In considering the CCBHC and Massachusetts CBHC models, several important observations emerge. The first is that they share more similarities than differences. Central to both models is a comprehensive outpatient mental health clinic for both children and adults, with multidisciplinary services, including not only psychotherapy and medication management but also case management, care coordination, peer services, supports for families and caregivers, and others. Both clinic models have a requirement to provide basic primary care services particularly focused on medical screening and basic physical health monitoring and coordination. A bundled payment rate, daily or monthly for CCBHCs and per-diem for CBHCs, is also the provider payment mechanism for both outpatient models, which enables coverage for services that typically fall outside fee-for-service reimbursement. Both models emphasize support around the social determinants of health (referred to in the Massachusetts CBHC model as health-related social needs). Both models include 24/7 crisis services, albeit with different models of care, and include community-based mobile crisis teams. Provider payment for these services is also separate from the daily rate in the Massachusetts CBHC model and in the CC PPS-3 and CC PPS-4 options of the CCBHC model. Finally, both models include a pay-for-performance financial incentive through structured clinical quality measures.

Beyond these basic similarities, several differences stand out. From a clinical standpoint, the exact scope of the required integrated mental health and substance use services is slightly different between CCBHCs and Massachusetts CBHCs. For example, the CCBHC model explicitly specifies a requirement to provide behavioral health services for veterans and individuals in the armed forces while the MA CBHC program does not. These priorities may reflect particular domains of unmet need across the US that are well suited to be addressed in the CCBHC setting. The Massachusetts CBHC model, on the other hand, focuses on rapid access, specifying timeframes by which services need to be provided—in some cases, same- or next-day. The Massachusetts model also specifies in greater detail specific therapeutic modalities that must be offered to patients, such as Dialectical Behavior Therapy and Cognitive Behavior Therapy. Massachusetts CBHCs also include the community crisis stabilization level of care, a 24/7 unit meant to represent an alternative to inpatient psychiatric hospitalization, of which there is no equivalent in the federal CCBHC model. These features may both reflect the period in which the Massachusetts CBHC model was developed—in the setting of high volumes of demand for inpatient psychiatric hospitalization during the COVID-19 pandemic—and the ability for a state-level agency to be more prescriptive around the precise clinical model. As another example, the Massachusetts CBHC model explicitly includes details about youth services, including youth mobile crisis intervention and youth community crisis stabilization services. This reflects that youth mental health was a particularly high priority for state and community stakeholders at the time of the CBHC model development.

In regard to provider payment, the federal CCBHC demonstration program is grounded in a cost-based Prospective Payment System (PPS) similar to that used to reimburse Federally Qualified Health Centers. A PPS model provides more volume-agnostic reimbursement that assures predictable funding tied to costs, enabling investment in necessary but traditionally non-billable services as noted above. The Massachusetts CBHC per-diem “encounter bundle” is similar, though not prospective. Furthermore, the Massachusetts CBHC rates are uniform across the state rather than clinic-specific. Although the necessity of a PPS with a cost-based rate is clearly greater at the national level, given the enormous variations in operating clinical programs between different regions of the US, variation is likely to be seen even between different parts of Massachusetts. An additional factor to consider in clinic-specific PPS rates is the actuarial expenses associated with developing reimbursement rates that align with actual service costs. Cost-based rate setting requires extensive analysis that may be administratively burdensome and require ongoing data collection and periodic adjustments based on continuing cost data.

The enhanced federal match rate for CCBHCs (at the CHIP-equivalent eFMAP of 65% or higher) presents a powerful financial incentive for states to adopt the federal demonstration model. By covering a larger percentage, the CCBHC demonstration program offers states the opportunity to bolster their mental health systems further. Massachusetts relies on the standard 50% federal match for most CBHC services. Although Massachusetts had been one of the 24 states chosen to participate in the original one-year CCBHC planning grant, it was not selected as a demonstration state thereafter. The state instead opted to forge its own path through the Roadmap for Behavioral Health Reform initiative and 1,115 waiver authorities.

The federal CCBHC demonstration has established Quality Bonus Payments (QBPs) that reward clinics meeting state-established benchmarks for previously determined quality measures; Massachusetts's CCQI and CQEIP similarly encourage providers to reach performance targets. However, there are substantial differences in how quality is measured between the two models. The CCBHC QBP program includes both a required measure on diabetes control and a required measure on depression symptom remission, based on patient-reported outcomes. These are alongside other required measures on timeliness of access, post-acute access, and readmissions, which share similarities with the Massachusetts CCQI. The CCBHC QBP reinforces a broader clinical mission, including a medical or primary care lens, whereas the Massachusetts CCQI focuses CBHCs on addressing urgent mental health needs and serving as the “front door” to behavioral health services. The Massachusetts CQEIP, by contrast, diverges substantially from the CCBHC QBP program and demonstrates the state's emphasis on equitable access to care, addressing social determinants of health, and cultural competence.

As other states consider developing a community behavioral health program, these comparative findings suggest that these decisions should be oriented toward fitting with individual statutory authority, delivery system capacity, and equity priorities rather than a presumption that one model is categorically superior. The interaction between these models underscores the diverse pathways states can take toward expanding comprehensive behavioral health care. Some states follow the CCBHC demonstration program and leverage the higher federal match, while others experiment with alternatives like 1,115 waivers, state plan amendments, or a blend of grant funding and state-led initiatives. Alignment with the federal CCBHC framework can expedite implementation through defined certification standards, technical support, and national comparability, though at the cost of reduced programmatic flexibility, additional reporting and compliance demands, and monetary costs to develop clinic-individualized, actuarially sound rates. A state-specific approach, such as the one seen in Massachusetts, may enable closer alignment with existing infrastructure, contracting arrangements, and population needs, while requiring greater design and implementation capacity. Massachusetts' choice to implement CBHCs demonstrates the importance of aligning new outpatient models with broader state reform efforts—namely, its Behavioral Health Roadmap —and to ensure feasibility of new models for its existing network of mental health providers. Local or state-level initiatives may have greater ability to tailor programs to the specific needs of their communities and incorporate input from community members. Regardless of the chosen route of development of a behavioral health clinic program, state regulators would benefit from preemptively developing a clear program intention and roadmap, building a set of statewide, minimum standards applicable across all payers, establishing early data specifications and technical assistance programs, and phasing implementation to support providers.

Notably, early findings from the federal demonstration program show some initial promise, including reductions in some states of acute behavioral health care utilization and increases in evidence-based practices provided, goals aligned with the fundamental goals of the Massachusetts CBHC model. Outcomes of the Massachusetts model, to be obtained after greater time has passed and data are collected, and comparisons to outcomes of the CCBHC model will be important to compare against their respective clinical components, provider reimbursement, and other policies. As these programs continue to be evaluated, policymakers in Massachusetts and other states will glean insight into which elements—enhanced match rates, cost-based reimbursement, strong crisis services, and quality incentives—are most impactful in improving mental health and substance use outcomes.

## Appendix 1: Glossary

**American Rescue Plan Act (ARPA)**: A law signed in March 2021, considered to build upon the Coronavirus Aid, Relief, and Economic Security Act (CARES Act), designed to provide broad relief and economic stimulus to the United States as it worked to recover from the economic and public health impacts of the COVID-19 pandemic.

**Centers for Medicare and Medicaid Services (CMS)**: The federal agency within the US HHS that administers Medicare, Medicaid, CHIP, and the Health Insurance Marketplace and sets many national healthcare payment and quality policies.

**Certified Community Behavioral Health Clinic (CCBHC)**: Outpatient-based program designed and regulated by the US federal government with the mandate to provide coordinated and comprehensive behavioral health care to all individuals who request care for mental health or substance use regardless of their ability to pay, place of residence, or age.

**Children's Health Insurance Program (CHIP)**: A joint federal–state program that covers eligible children and, in some states, pregnant people, in families with incomes too high for Medicaid but too low for commercial coverage.

**CBHC Clinical Quality Incentive Program (CCQI)**: A component of MassHealth's CBHC Incentive Program that pays CBHCs based on performance on defined clinical quality measures.

**Community Behavioral Health Center (CBHC)**: Outpatient-based clinics and crisis services designed and regulated by Massachusetts EOHHS with the mandate of providing coordinated and comprehensive behavioral health care to all individuals who request care for mental health or substance use regardless of their ability to pay, place of residence, or age.

**Coronavirus Aid, Relief, and Economic Security Act (CARES Act)**: A law signed in 2020 designed to provide economic stimulus and relief to the United States in response to the COVID-19 pandemic.

**Enhanced Federal Medical Assistance Percentages (eFMAP)**: A higher-than-regular federal matching rate applicable to certain Medicaid/CHIP expenditures, set in statute as an enhancement over the state's base FMAP.

**Executive Office of Health and Human Services (EOHHS)**: The Massachusetts Governor's cabinet-level secretariat that oversees MassHealth and other human-services departments, coordinating health and social services across Massachusetts.

**Federal Medical Assistance Percentage (FMAP)**: The federal share of Medicaid service costs in each state, calculated via a formula as the inverse to state per-capita income.

**MassHealth**: Massachusetts’ combined Medicaid and Children's Health Insurance Program (CHIP) agency, providing health care coverage for care to eligible children, families, seniors, and people with disabilities in the Commonwealth, administered by the Massachusetts EOHHS.

**Prospective Payment System (PPS)**: A reimbursement method where payment is calculated as a predetermined, fixed amount, often per stay, per visit, or per episode, rather than based on individual services, such as in a fee-for-service system.

**Protecting Access to Medicare Act (PAMA)**: A federal law that, among other provisions, authorized the CCBHC demonstration.

**CBHC Quality and Equity Incentive Program (CQEIP)**: A component of MassHealth's CBHC Incentive Program that pays CBHCs for improvements in health equity, demographic data collection, and reductions in health care related disparities.

**Quality Bonus Payments (QBPs)**: Performance-based payments tied to quality results.

**Special Crisis Services (SCS)**: In the Massachusetts CBHC model, crisis services including mobile crisis intervention and community crisis stabilization paid separately from the outpatient encounter bundle.

**Special Population (SP)**: Groups with distinct clinical or eligibility needs (e.g., medically frail adults, people with disabilities, dual-eligible individuals) in the CCBHC program who may receive tailored benefits, care coordination, or payment adjustments to account for their increased complexity or greater acuity.

**Substance Abuse and Mental Health Services Administration (SAMHSA)**: The HHS agency that leads national efforts to improve behavioral health, prevent substance use, and support treatment and recovery.

**US Department of Health and Human Services (HHS)**: The federal cabinet department which houses federal agencies tasked with enhancing the health of Americans.
